# Experimental Inoculation in Rats and Mice by the Giant Marseillevirus Leads to Long-Term Detection of Virus

**DOI:** 10.3389/fmicb.2018.00463

**Published:** 2018-03-21

**Authors:** Sarah Aherfi, Claude Nappez, Hubert Lepidi, Marielle Bedotto, Lina Barassi, Priscilla Jardot, Philippe Colson, Bernard La Scola, Didier Raoult, Fabienne Bregeon

**Affiliations:** ^1^Institut Hospitalo Universitaire Méditerranée Infection, Assistance Publique-Hôpitaux de Marseille, Centre Hospitalo Universitaire Timone, Pôle des Maladies Infectieuses et Tropicales Clinique et Biologique, Fédération de Bactériologie-Hygiène-Virologie, Marseille, France; ^2^Laboratoire d'Anatomopathologie, Centre Hospitalo Universitaire Timone, Assistance Publique des Hôpitaux de Marseille, Marseille, France; ^3^Service des Explorations Fonctionnelles Respiratoires Centre Hospitalo Universitaire Nord, Pôle Cardio-Vasculaire et thoracique, Assistance Publique des Hôpitaux de Marseille, Marseille, France

**Keywords:** marseillevirus, experimental infection, murine model, giant viruses, *Megavirales*, NCLDV, pathogenicity

## Abstract

The presence of the giant virus of amoeba Marseillevirus has been identified at many different sites on the human body, including in the bloodstream of asymptomatic subjects, in the lymph nodes of a child with adenitis, in one adult with Hodgkin's disease, and in the pharynx of an adult. A high seroprevalence of the Marseillevirus has been recorded in the general population. Whether Marseillevirus can disseminate and persist within a mammal after entry remains unproven. We aimed to assess the ability of the virus to disseminate and persist into healthy organisms, especially in the lymphoid organs. Parenteral inoculations were performed by intraperitoneal injection (in rats and mice) or intravenous injection (in rats). Airway inoculation was performed by aerosolization (in mice). Dissemination and persistence were assessed by using PCR and amebal co-culture. Serologies were performed by immunofluorescent assay. Pathological examination was conducted after standard and immunohistochemistry staining. After intraperitoneal inoculation in mice and rats, Marseillevirus was detected in the bloodstream during the first 24 h. Persistence was noted until the end of the experiment, i.e., at 14 days in rats. After intravenous inoculation in rats, the virus was first detected in the blood until 48 h and then in deep organs with infectious virus detected until 14 and 21 days in the liver and the spleen, respectively. Its DNA was detected for up to 30 days in the liver and the spleen. After aerosolization in mice, infectious Marseillevirus was present in the lungs and nasal associated lymphoid tissue until 30 days post inoculation but less frequently and at a lower viral load in the lung than in the nasal associated lymphoid tissue. No other site of dissemination was found after aerosol exposure. Despite no evidence of disease being observed, the 30-day long persistence of Marseillevirus in rats and mice, regardless of the route of inoculation, supports the hypothesis of an infective potential of the virus in certain conditions. Its constant and long-term detection in nasal associated lymphoid tissue in mice after an aerosol exposure suggests the involvement of naso-pharyngeal associated lymphoid tissues in protecting the host against environmental Marseillevirus.

## Introduction

Giant viruses of amoebas were discovered in 2003, with the isolation of *Acanthamoeba polyphaga* Mimivirus by co-culturing on amoeba. *Marseilleviridae* is a new family of amoebal giant viruses defined in 2012 (Colson et al., 2013b). Its founding member is Marseillevirus (Boyer et al., 2009), and in addition 12 other members have been described to date including Senegalvirus, Cannes 8 virus, Fontaine Saint Charles virus, Melbournevirus, Lausannevirus, Tokyovirus, Tunsivirus, Insectomime virus, Brazilian Marseillevirus, Golden Marseillevirus, and Port-Miou virus (Boyer et al., 2009; La Scola et al., 2010; Thomas et al., 2011; Lagier et al., 2012; Aherfi et al., 2013, 2014; Boughalmi et al., 2013; Doutre et al., 2014, 2015; Dornas et al., 2016; Takemura, 2016). Subsequently, contact between giant viruses and humans were suggested. Concordant data argue for the pathogenicity of these viruses, such as mimiviruses-associated pneumonia (La Scola et al., 2005; Raoult et al., 2006; Bousbia et al., 2013; Saadi et al., 2013a,b) or the recently described association between phycodnaviruses and cognitive impairment (Yolken et al., 2014). The presence of giant viruses of amoeba, including those of marseilleviruses within human biological material, was more recently revealed by high throughput metagenomics, confirming contacts between these viruses and humans (Colson et al., 2013a; Rampelli et al., 2016; Verneau et al., 2016).

Senegalvirus was the first marseillevirus to be isolated from human samples, following its serendipitous detection during a microbial metagenomic study conducted on the stools of a healthy Senegalese man (Lagier et al., 2012). In 2013, a metagenomic study further revealed the presence of a substantial number of reads matching the Marseillevirus genome in the viral fraction of healthy blood donors (Popgeorgiev et al., 2013a). Hypotheses were then generated around blood carriage and the blood-borne transmission of Marseillevirus. Furthermore, two seroprevalence studies unexpectedly suggested frequent contacts between humans and Marseillevirus (Mueller et al., 2013; Popgeorgiev et al., 2013b). The detection of giant viruses of amoebae in humans in association with clinical symptoms may be coincidental, but this is nevertheless an emerging issue. A single clinical observation has reported the detection of a marseillevirus in a pathological lymph node of a 11-month-old boy with lymphadenitis (Popgeorgiev et al., 2013c). We subsequently reported the presence of Marseillevirus in the blood and lymph nodes of a patient with Hodgkin's disease (Aherfi et al., 2016a). We also detected Marseillevirus DNA by PCR in two pharyngeal samples collected from a 20-year-old patient presenting neurological disorders at a one-year interval, strongly suggesting the viral persistence of this agent in the tonsils (Aherfi et al., 2016b). To date, no data argued for Marseillevirus propagation in mammal cells and no causal relationship has been established between the presence of this virus and clinical symptoms or diseases observed in these different cases. The only one known host of Marseillevirus that allows a complete lytic cycle is Acanthamoeba cells. To our knowledge, the study of viruses in non host organisms and their interaction remain an unexplored area of virology. Taken together, these findings suggest however that the particles or DNA markers of Marseillevirus may persist during a long period in humans in some cases. Such a hypothesis requires further experimental data.

With this goal, we set up a murine model using rats and mice and different routes of inoculation to assess the dissemination and the persistence of Marseillevirus in mammalian organisms. The aerosol route, we think plausible route of transmission of this waterborne virus (Boyer et al., 2009), was tested first with a special focus on the localization and persistence of the virus in the nasal associated lymphoid tissue (NALT) as an equivalent to the human tonsils. We also tested the intraperitoneal and the intravenous routes in mice and rats.

## Materials and methods

### Ethics statements and general procedures *in vivo*

For animal studies, the experimental protocols, registered by the “Ministère de l'Enseignement Supérieur et de la Recherche” under reference number 20150528122362 and 2015060517005844, were approved by the Institutional Animal Care and Use Committee of Aix-Marseille University “C2EA-14,” France. We used Balb/c mice between 4 and 8 weeks old (Envigo Laboratories, Gannat, France) weighing between 16 and 25 g, and Swiss rats weighing between 330 and 770 g. Animals were housed in individual plastic cages (five mice or two rats per cage) in a ventilated pressurized cabinet (A-BOX 160, Noroit, Rezé, France) with free access to water and standard diet food until the experiment. All animals were housed in protected environmental area and received standard diet including dehydrated rodent feed pellets and sterile water.

Airway inoculation was performed by aerosol delivery using the whole-body inhalation exposure system A4224 (IES, Glas-Col LLC, Terre Haute, USA). Intraperitoneal (IP) and intravenous (IV) inoculations were performed under volatile anesthesia with 5% isoflurane, by percutaneous puncture of the abdomen or injection into the tail vain, respectively.

Control animals received phosphate buffered saline (PBS) via the aerosolized, IP or IV routes according to the same time of exposure or the same volume as infected animals. After inoculations, the animals were transferred into cages and housed in a safety cabinet with food and water *ad libitum*.

Serial blood samples were taken from the IV injected rats over time by tail vein puncture to describe the kinetics of viremia. At the end of the experiments, the rats were euthanized with a lethal dose of thiopental (Panpharma, France) administered intraperitoneally and the mice were euthanized with exsanguination performed under volatile anesthesia. Additional blood and organ samples were collected post-mortem.

### Strains, culture conditions, and preparation of infective inoculums for animal experiments

Marseillevirus strain T19 was co-cultured on axenic *Acanthamoeba castellanii*, in peptone yeast extract broth with glucose medium (PYG). The culture supernatants were then concentrated and purified as previously described and finally washed in PBS (Dornas et al., 2015). The purified virus was aliquoted and stored at −80°C for further use. Ten days before the animals were inoculated, the viable virus was quantified by end point dilution by co-culturing on *A. castellanii*. At this end, serial dilutions of the virus suspension with a dilution factor of 10 were inoculated to amoebas at a concentration of 5.10^5^/mL deposited in a 24 well plate. Amoebas were inoculated with each dilution of virus in quadruplicate. The amoeba were checked for lysis 7 days after inoculation. The concentration of the viable virus was those that allowed the amoeba lysis in two wells of the four that were inoculated with this concentration. The concentration of purified viable virus ranged between 7 and 7.5 log units per μL. Purified viruses were diluted in PBS immediately before inoculating the animals, to reach the appropriate inoculums (see “Animal experiments”).

### Inoculation of marseillevirus *in vivo*

For airway inoculation, 81 mice (34 males, 47 females) were aerosol-inoculated with a suspension of PBS containing nine log units of viruses per mL placed into the glass vial for liquid venturi aerosol generation following the manufacturer's recommendations and custom settings. As assessed on animals euthanized just after aerosol exposition (*n* = 4), the initial viral lung inoculum ranged between 4.9 and 5.7 (mean 5.6) log units of viral copies per million murine cells.

For the parenteral inoculations, eight log units of viable virus diluted in 300 μL of PBS were injected to 21 rats IV and 12 rats IP. For mice, seven log units of viable virus diluted in 200 μL of PBS were injected IP (*n* = 15).

### Follow-up and samplings

After the inoculations, the animals were observed daily for signs of discomfort or illness.

The IP route was assessed for 24 h in mice and until 2 weeks post-inoculation (PI) in a series of rats euthanized at 12 h, and at days 1, 3, 7, 14, and 43 PI. The IV route in rats was assessed until day 43 PI, with evaluation taking place at 12 h, and on days 1, 2 (blood only), 3, 7, 14, 21, 30, and 43 PI. The aerosol route in mice was assessed until 1 month PI with evaluation taking place at 2 h, and on days 1, 7, 14, 21, and 30 PI.

For all animals, the spleen, liver, and blood were collected. In addition, the omentum and mesenteric lymph nodes from IP inoculated rats, the cervical lymph nodes from IV inoculated rats, the lungs, the NALT, and the cervical and tracheal lymph nodes from aerosol inoculated mice were sampled immediately *post mortem*. Spleen weight was immediately recorded and blood was aliquoted for PCR and serology.

To avoid detecting the possible contamination of the external organ with the virus due to the IP inoculation process, the removed abdominal organs were decontaminated in two baths of 70° ethanol and then washed in PBS before culture and PCR processing.

Each freshly sampled organ was separately crushed in PBS for amoebal co-culture and DNA extraction was performed for PCR.

Representative samples of lungs, lymph nodes, NALT, spleen and liver from each evaluation time were fixed in 4% formalin for histological analyses, including a total of 10 spleen and liver samples, 11 lung samples, and 12 NALT samples from aerosol inoculated mice.

### Amoebal co-culture

**Figure 1:**
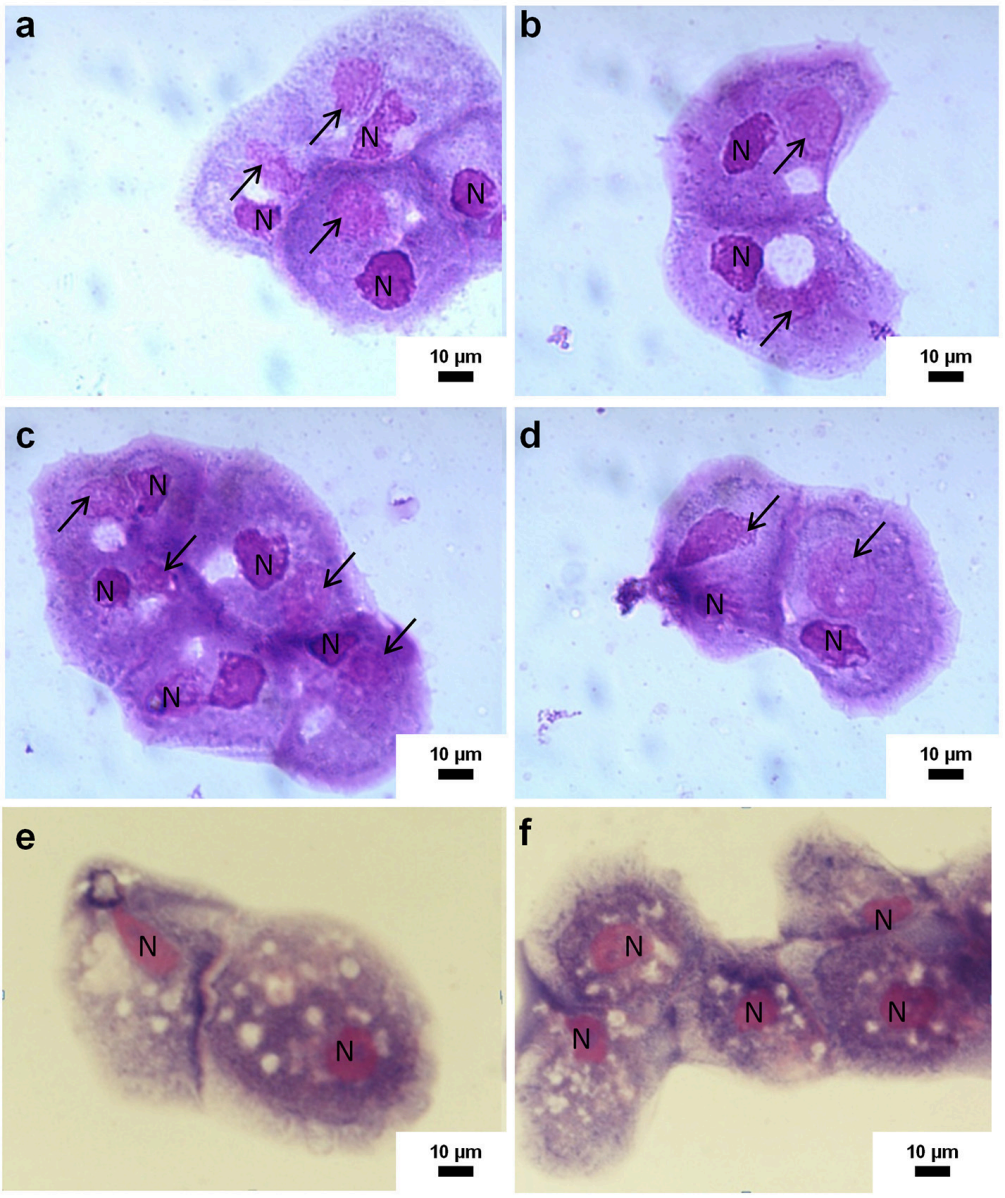
Representative photomicrographs of co-culture of mice and rats samples on *Acanthamoeba castellanii*. Pictures **(a–d)** show positive samples by co-culture as indicated by the visible infected amoebas with Marseillevirus. These samples include NALT from aerosol inoculated mice and spleen from rats after IV inoculation with Marseillevirus. The arrows indicate viral factories and N indicate the nucleus of amoeba. Pictures **(e,f)** show negative samples by amoebal co-culture: the amoebas are not infected and do not contain viral factories.

*A. castellanii* was cultured at 28°C in PYG. When amoebas were confluent, they were centrifuged, and the pellet was resuspended in sterile Page's amoeba saline solution twice. Finally, amoebas were resuspended in survival buffer solution at a final concentration of between 5.10^5^ and 1.10^6^ cells/mL with an antimicrobial mix consisting of imipenem / cilastatin (10 μg/mL), vancomycin (10 μg/mL), ciprofloxacin (20 μg/mL), doxycycline (20 μg/mL), and voriconazole (20 μg/mL). Amoebas were distributed in 24-well plates (500 μL of amoeba culture per well). 50 μL of each of the crushed organs was then deposited on the cell layer and incubated at 30°C for 3 days. Two sub-cultures were performed. When amoeba lysis occurred, 100 μL of the well content was spotted on a slide and colored using hemacolor staining (Hemacolor®, Merck, Darmstadt, Germany) to check for the presence of viral factories (Boughalmi et al., 2012; Figure 1). Wells containing only amoebas were included in each microplate as negative controls.

### Molecular detection of marseillevirus

The DNA from the total blood and from the crushed organs was extracted using a QIAamp Tissue Kit (Qiagen). Two systems of specific primers and probes were used for quantitative real-time PCR (reg4-2-F: CCCAACAGAGGCCGAAATT, reg 4-2R: CCTTCTGTACGAGGCCAAAA, probe reg4-2: TCCTCCCCAGAACCAGACTCTCCA, reg 8-2 F: TCTTGTCTGGCTTTCCCTTC, reg8-2 R: GTGTCTCTGCCTGTCCAAA, probe reg8-2: AGTGAGGAGTCTGTTGGCCGCA). These two systems target specifically MAR_ORF210 (encoding a hypothetical protein excluded from Genbank database due to the lack of start codon) and MAR_ORF055 (encoding a RNA polymerase Rpb1 domains 1-2), respectively. These two genes are in single copies in the Marseillevirus strain T19 genome. When an amplification was obtained and a fluorescence signal was generated by testing both the two systems of PCR, the result was considered as positive if the cycle threshold was <35 for at least one of the two systems.

When amplification was obtained and a fluorescent signal was generated with only one of the two systems, whichever the cycle threshold, the result was considered as negative. The amplification of housekeeping genes hydroxymethylbilane synthase and glyceraldehyde-3-phosphate dehydrogenase were used as internal controls for mice and rats, respectively (Huang et al., 2008; Ding et al., 2014).

Real time PCR assays were performed using the CFX96® qPCR Detection System (Bio-Rad, France). Negative controls consisted of DNA extracted from the organs and blood of PBS-challenged mice and rats (two animals for each route of inoculation). Positive controls were DNA extracted from Marseillevirus culture supernatants.

Viral loads were calculated on the basis of the calibration standard curve of DNA from a suspension of purified Marseillevirus, the concentration of which was determined by flow cytometry (Brussaard, 2004). To standardize the amounts, the viral loads into the tissues were expressed as n log units of viral copies per million murine cells.

### Immunofluorescence assay for marseillevirus antibodies detection in sera

In the aim to have positive controls for serological tests on the rats and mice of the experiments, we previously immunized a rabbit with Marseillevirus by the subcutaneous route. After three inoculations, serum from the rabbit consisting in polyclonal antibodies specific to Marseillevirus, was collected and used as a positive control for serological testing.

Purified Marseillevirus was spotted on microscope slides. Sera collected from rats and mice were tested at the 1:50 dilution in PBS. Sera were deposited on the spots and incubated 30 min at 37°C. Slides were washed twice in PBS/Tween20 0.5% during 8 min, once in distilled water during 8 min, then dried. The presence of antibodies was detected using a FITC (fluorescein isothiocyanate) conjugated goat anti-mouse IgG (Immunotech, Marseille, France), anti-mouse IgM at 1: 400 dilution (Jackson Immunoresearch Laboratories, West Grove, USA) for mouse sera, and anti-rat IgG (Jackson ImmunoResearch, Suffolk, United Kingdom) for rat sera, with Evans blue counterstain 0.25%. Slides were incubated at 37°C during 30 min, washed twice in PBS/Tween 20 0.5% during 8 min, once in distilled water during 8 min, then dried. The slides were then observed after adding 1 drop of Fluoprep (Biomérieux, France) and coverslips, on a microscope Leica DM 2,500 (Leica, Wetzlar, Germany) at 488 nm wavelength. As negative controls, sera from non-immunized mice were included in each experiment. Positive controls consisted in sera from immunized rabbit. The threshold for positivity of serology was the 1:50 dilution of the mice and rat sera. A result was considered as positive if the two observers, blind to group assignment, so concluded. Any discordant result was considered as negative.

## Results

### Dissemination of the virus

In IP inoculated rats and mice, dissemination of the virus into the bloodstream was observed at day 1 PI, as attested by positive PCR in two of the four rats and two of the four mice tested (Supplementary Files 1, 2). The mean viral loads were at day 0, 3.9, and 6.1 and at day 1, 3.9, and 8 log units per million murine cells, respectively in rats and mice. Blood samples induced amoeba lysis in 5/7 of the rat and mice blood samples collected at day 0 and 2/8 at day 1 PI. Dissemination of the viable virus to deep organs was also observed in the spleen and liver (see below “Persistence of viruses”).

**Figure 2:**
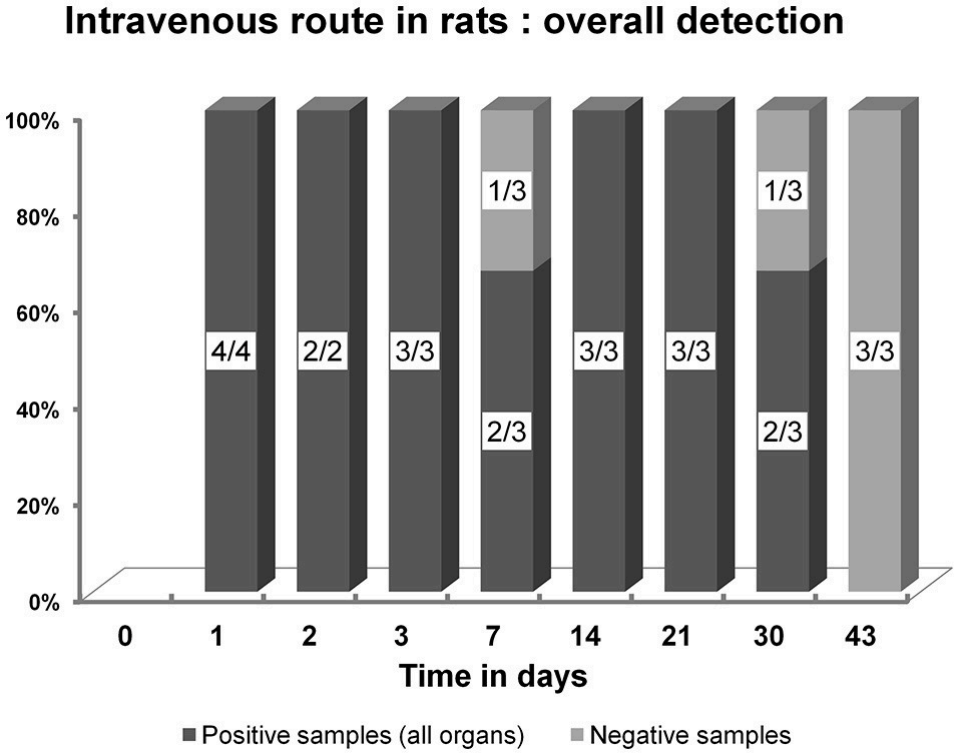
Overall detection and persistence of Marseillevirus DNA by PCR after an intraveinous inoculation in rats, during the 43 day long experiment.

As expected, after IV inoculation in rats, the virus was detected in blood samples, but also in deep organs as attested by amoeba co-culture and PCR (Tables 1, 2, Figure 2, Supplementary File 3). At 24 h PI, the liver, spleen and lungs were found positive for all the rats tested (4/4).

**Table 1:** Summary of results obtained by qPCR of blood and organs of rats and mice inoculated with Marseillevirus.

**PCR**			**Day 0**	**Day 1**	**Day 2**	**Day 3**	**Day 7**	**Day 14**	**Day 21**	**Day 30**	**Day 43**
IP route	Rats	Blood	Positive 33.0 (*n* = 2/3)	Positive 33.3 *(n* = 1/4)	ND	Negative - (*n* = 0/2)	Negative - (*n* = 0/4)	Negative - (*n* = 0/3)	ND	ND	NI
		Spleen	ND	Positive 33.7 (*n* = 2/2)	ND	Positive 34.9 (*n* = 1/2)	Positive 34.7 (*n* = 3/3)	Positive 33.6 (*n* = 3/3)	ND	ND	Negative - (*n* = 0/1)
		Liver	ND	Positive 33.3 (*n* = 1/2)	ND	Positive 34.9 (*n* = 1/2)	Negative (*n* = 0/2)	Positive 33.7 (*n* = 2/3)	ND	ND	Negative - (*n* = 0/1)
	Mice	Blood	Positive 31.8 (*n* = 2/4)	Positive 31.7 (*n* = 2/4)	Positive 32.0 (1/3)	ND	Negative - (*n* = 0/3)	ND	Negative - (*n* = 0/1)	ND	ND
		Spleen	Positive 31.7 (*n* = 3/4)	Positive 33.2 (*n* = 2/4)	Positive 34.2 (*n* = 1/3)	ND	Positive 31.0 (*n* = 1/3)	ND	Negative - (*n* = 0/1)	ND	ND
		Liver	ND	ND	ND	ND	ND	ND	ND	ND	ND
IV route	Rats	Blood	ND	Positive 33.2 (*n* = 4/4)	Positive 32.8 (*n* = 2/2)	Negative - (*n* = 0/2)	Negative - (*n* = 0/3)	Negative - (*n* = 0/3)	Negative - (*n* = 0/3)	Negative - (*n* = 0/3)	Negative - (*n* = 0/2)
		Spleen	ND	Positive 29.4 (*n* = 4/4)	ND	Positive 31.1 (*n* = 3/3)	Positive 29.0 (*n* = 2/3)	Positive 31.5 (*n* = 3/3)	Positive 28.2 (*n* = 2/2)	Positive 28.6 (*n* = 2/3)	Negative - (*n* = 0/2)
		Liver	ND	Positive 29.2 (*n* = 4/4)	ND	Positive 29.0 (*n* = 3/3)	Positive 31.0 (*n* = 1/3)	Positive 30.2 (*n* = 3/3)	Positive 28.2 (*n* = 2/2)	Positive 29.1 (*n* = 2/3)	Negative - (*n* = 0/2)
Aerosol route	Mice	Blood	Negative - (*n* = 0/4)	Negative - (*n* = 0/15)	ND	ND	Negative - (*n* = 0/17)	Negative - (*n* = 0/15)	Negative - (*n* = 0/12)	Negative - (*n* = 0/5)	ND
		Spleen	Negative - (*n* = 0/4)	Negative - (*n* = 0/15)	ND	ND	Negative - (*n* = 0/18)	Negative - (*n* = 0/15)	Negative - (*n* = 0/14)	Negative - (*n* = 0/5)	ND
		Liver	Negative - (*n* = 0/4)	Negative - (*n* = 0/15)	ND	ND	Negative - (*n* = 0/18)	Negative - (*n* = 0/15)	Negative - (*n* = 0/14)	Negative - (*n* = 0/5)	ND
		Lung	Positive 26.1 (*n* = 4/4)	Positive 28.4 (*n* = 15/15)	ND	ND	Positive 30.7 (*n* = 16/18)	Positive 34.6 (*n* = 8/15)	Negative - (*n* = 0/14)	Negative - (*n* = 0/4)	ND
		NALT	Positive 29.7 (*n* = 2/2)	Positive 31.0 (*n* = 11/15)	ND	ND	Positive 31.5 (*n* = 18/18)	Positive 33.6 (*n* = 13/15)	Positive 34.8 (*n* = 7/14)	Positive 33.2 (*n* = 3/3)	ND

*Mean Ct obtained by qPCR for IP route and aerosol routes in mice, IP and IV routes in rats. In each case, the first line is the result: positive / negative, the second line is the mean Ct obtained for the positive samples tested, the third line is the number of positive samples/number of tested samples. ND, Not Done*.

**Table 2:** Summary of results obtained by amoebal co culture of blood and organs of rats and mice inoculated with Marseillevirus.

**Coculture**			**Day 0**	**Day 1**	**Day 2**	**Day 3**	**Day 7**	**Day 14**	**Day 21**	**Day 30**	**Day 43**
IP route	Rats	Blood	Positive (*n* = 1/2)	Positive (*n* = 1/4)	ND	Negative (*n* = 0/2)	Negative (*n* = 0/4)	Negative (*n* = 0/3)	ND	ND	Negative (*n* = 0/1)
		Spleen	ND	Positive (*n* = 2/2)	ND	Positive (*n* = 1/2)	Positive (*n* = 3/4)	Positive (*n* = 3/3)	ND	ND	Negative (*n* = 0/1)
		Liver	ND	Positive (*n* = 2/2)	ND	Negative (*n* = 0/2)	Positive (*n* = 1/4)	Negative (*n* = 0/3)	ND	ND	Negative (*n* = 0/1)
	Mice	Blood	Positive (*n* = 3/4)	Positive (*n* = 1/4)	Negative (*n* = 0/3)	ND	Negative (*n* = 0/3)	ND	Negative (*n* = 0/1)	ND	ND
		Spleen	Positive (*n* = 4/4)	Positive (*n* = 4/4)	Positive (*n* = 2/3)	ND	Positive (*n* = 2/3)	ND	Negative (*n* = 0/1)	ND	ND
		Liver	ND	ND	ND	ND	ND	ND	ND	ND	ND
IV route	Rats	Blood	ND	Positive (*n* = 4/4)	Positive (*n* = 2/2)	Negative (*n* = 0/3)	Negative (*n* = 0/3)	Negative (*n* = 0/3)	Negative (*n* = 0/3)	Negative (*n* = 0/3)	Negative (*n* = 0/2)
		Spleen	ND	Positive (*n* = 4/4)	ND	Positive (*n* = 2/3)	Positive (*n* = 2/3)	Positive (*n* = 3/3)	Positive (*n* = 2/2)	Negative (*n* = 0/3)	Negative (*n* = 0/2)
		Liver	ND	Positive (*n* = 4/4)	ND	Positive (*n* = 3/3)	Positive (*n* = 2/3)	Positive (*n* = 2/3)	Negative (*n* = 0/2)	Negative (*n* = 0/3)	Negative (*n* = 0/2)
Aerosol route	Mice	Blood	Negative (*n* = 0/4)	Negative (*n* = 0/5)	ND	ND	Negative (*n* = 0/18)	Negative (*n* = 0/15)	Negative (*n* = 0/14)	Negative (*n* = 0/5)	ND
		Spleen	Negative (*n* = 0/4)	Negative (*n* = 0/5)	ND	ND	Negative (*n* = 0/18)	Negative (*n* = 0/15)	Negative (*n* = 0/14)	Negative (*n* = 0/5)	ND
		Liver	Negative (*n* = 0/4)	Negative (*n* = 0/5)	ND	ND	Negative (*n* = 0/18)	Negative (*n* = 0/15)	Negative (*n* = 0/14)	Negative (*n* = 0/5)	ND
		Lung	Positive (*n* = 4/4)	Positive (*n* = 15/15)	ND	ND	Positive (*n* = 16/18)	Positive (*n* = 8/15)	Positive (*n* = 1/14)	Positive (*n* = 1/4)	ND
		NALT	Positive (*n* = 2/2)	Positive (*n* = 11/11)	ND	ND	Positive (*n* = 18/18)	Positive (*n* = 14/15)	Positive (*n* = 11/14)	Positive (*n* = 3/3)	ND

*For some times of evaluation, the number of samples tested by coculture is different from those appearing in the table showing PCR results because of either insufficient quantity or ininterpretable results of PCR (negativity for internal control DNA and Marseillevirus DNA). In each case, the second line is the number of positive samples / number of tested samples. ND = Not done*.

**Figure 3:**
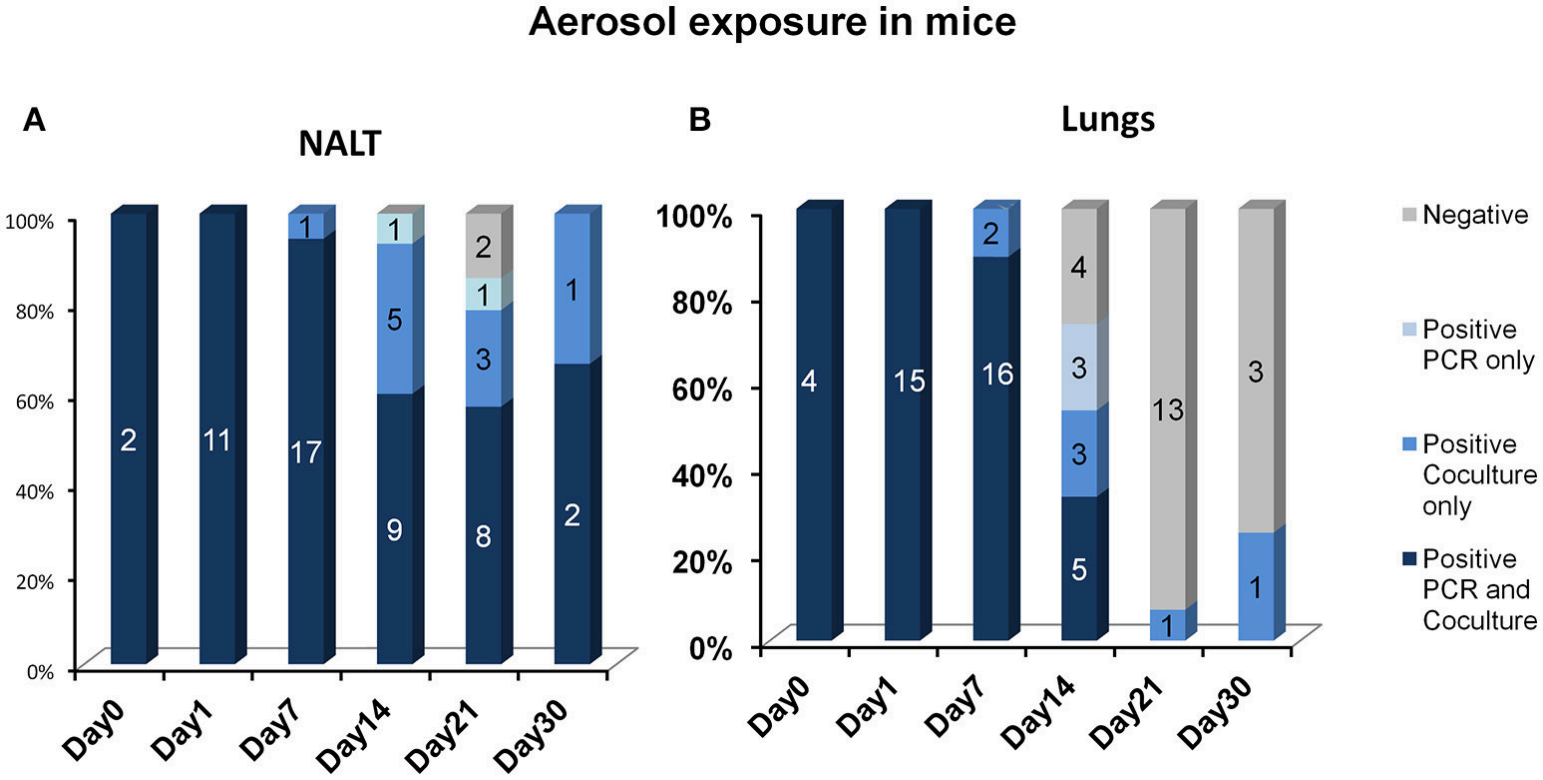
Positive **(A)** NALT and **(B)** lung samples, according to the technique (PCR or amoebal co-culture), after pulmonary inoculation. The percentage of positive samples is indicated on the y axis. The absolute numbers of positive samples are indicated by labels on the plots.

In aerosol inoculated mice, all animals had negative PCR and co-culture for the blood, spleen, liver and lymph nodes. In contrast, the lungs and NALT were frequently positive, regardless of the sample time, i.e., in 111 of 133 (83%) of the whole tested samples from aerosol inoculated mice, including 50 of 70 (71%) lung samples and 61 of 63 (97%) NALT samples (Figure 3, Supplementary File 4).

### Persistence of viruses

After IP inoculation, viable Marseillevirus i.e., detected by co-culture was detected in the spleen from the 12 h PI and persisted until the end of the experiment 14 days later (3/3 rats) (Supplementary File 2). In the liver and the omentum, the viable virus was recovered in the first seven days in rats, and viral DNA i.e., detected by PCR persisting up to day 14 in both organs.

**Figure 4:**
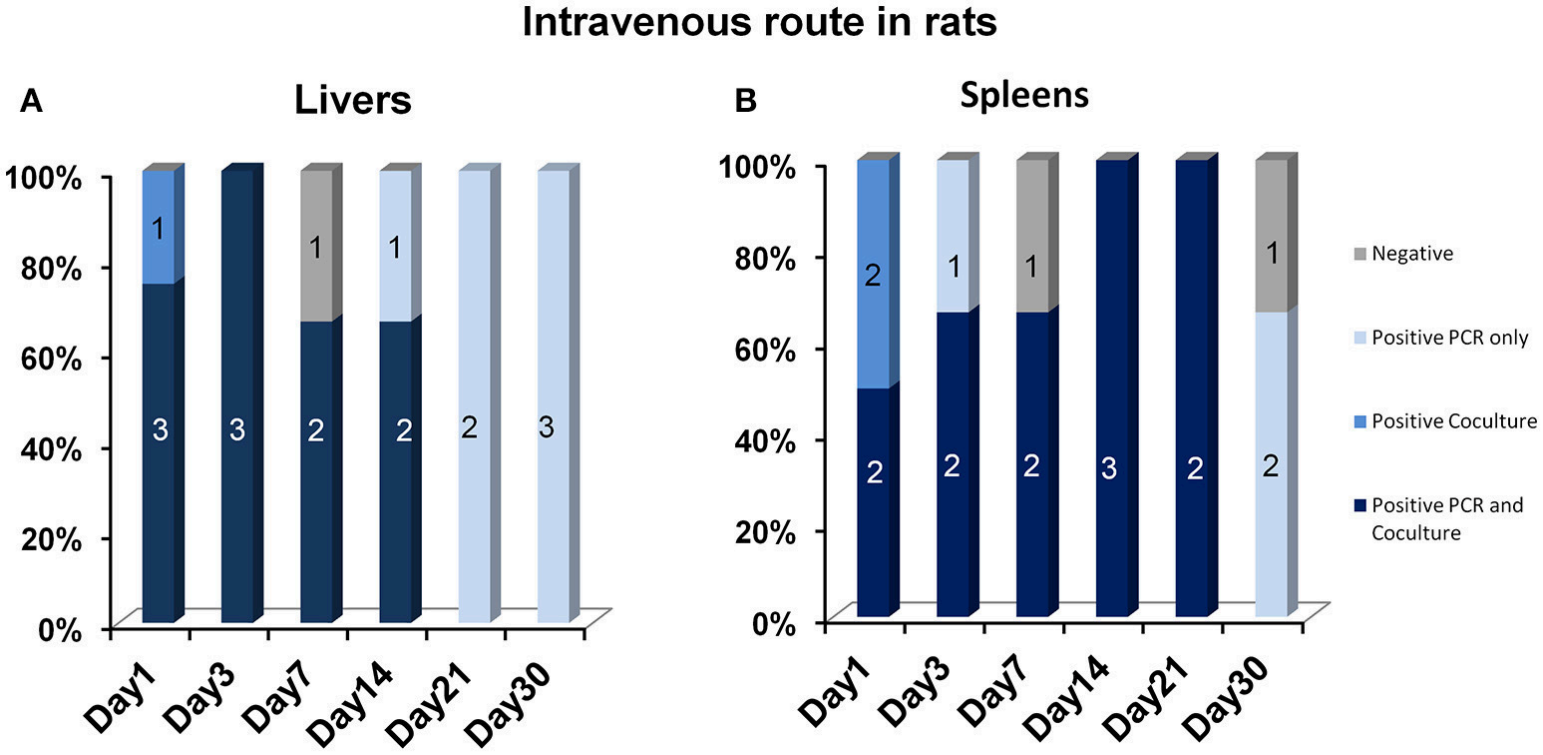
Positive **(A)** liver and **(B)** spleen samples, according to the technique (PCR or amoebal co-culture), after intravenous inoculation in rats. The percentage of positive samples is indicated on the y axis. The absolute numbers of positive samples are indicated by labels on the plots.

After IV inoculation in rats, the blood detection of Marseillevirus persisted up to 48 h PI in eight of the eight tested blood samples (PCR and culture). In the other organs, the viable virus was detected until days 14 and 21 in the liver and the spleen, respectively, while viral DNA persisted up to day 30 PI in both organs (Figure 4, Supplementary File 3).

**Figure 5:**
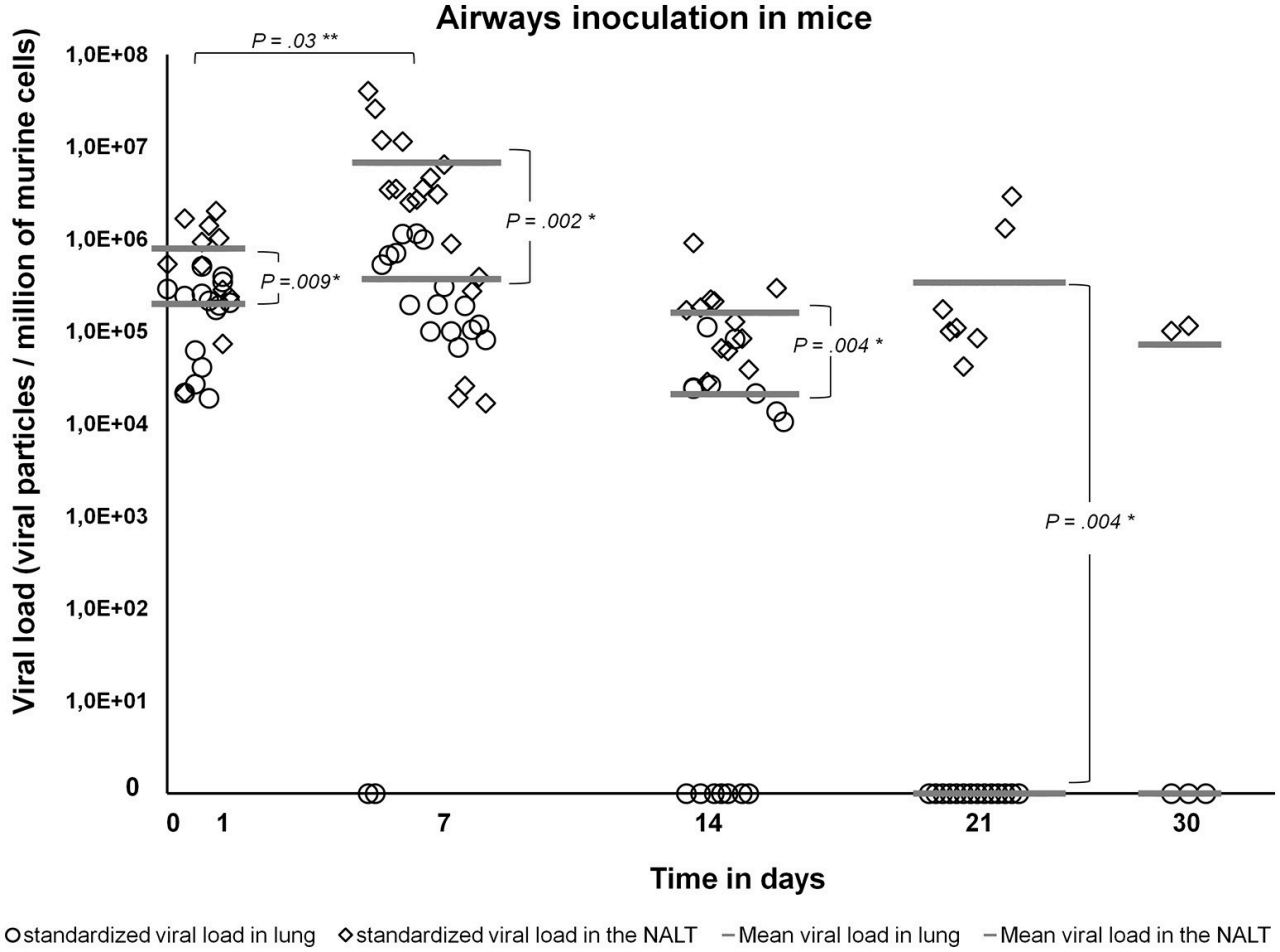
Standardized viral loads (viral copies per million murine cells) in the lungs and the NALT of aerosol-inoculated mice by Marseillevirus suspension. Diamonds represent the viral loads in the NALT and circles represent the viral loads in the lungs for each animal. The mean viral loads are represented for each time point by thick dashes. The viral loads in lungs and NALT, and at different time points were compared by using Wilcoxon-Mann Whitney test, using sigmaplot 13 SYSTAT software Inc. software. Single asterisks show the time points with significant differences between NALT and lung viral loads. Double asterisks show significant differences between the NALT viral loads at different time points.

After aerosolization, viable Marseillevirus persisted at least 30 days in the NALT in all the mice tested, and in only one lung sample of the four collected at the same time point (Figure 3, Supplementary File 4). Immediately after aerosolization and after 12 h post exposure, the viral load did not significantly differ between the NALT and the lung samples (*p* = 0.27). Between days 1 and 7 PI, the NALT viral loads increased. Moreover, from days 1 to 21 PI, the NALT viral loads were higher than in the lungs, then, despite decreasing, remained above the lung viral load (Figure 5).

### Serology

A total of 111 sera including nine from IP inoculated rats, 20 from IV inoculated rats and 82 from aerosol inoculated mice were tested.

After parenteral inoculation, of the eight sera collected between days 1 to 7 PI, two collected on day 7 PI, were positive for anti-Marseillevirus IgG. In IV inoculated rats, IgG anti-Marseillevirus antibodies were found in one of four sera collected on day 7 PI and 10 of 11 sera tested between days 14 and 43 PI. A representative microphotograph is presented in Supplementary File 5.

Only one aerosol inoculated mouse showed an IgG antibody response to Marseillevirus (sampled at day 30 PI).

All sera were negative for IgM, regardless of the inoculation route.

In eight cases, a positive signal was found by only one of the two observers. These samples were recorded as being negative. This concerned IgM antibodies on day 7 for two animals and day 16 for two others, and IgG at day 23 for four animals.

### Clinical outcome

No spontaneous deaths occurred and no animal presented signs of discomfort throughout the course of the experiment, whatever the route of virus inoculation. A regular gain in body weight occurred in all infected and control animals.

### Histopathological findings

No histological lesions were found in any murine tissue including NALT, the lungs, spleen, liver, cervical and tracheal lymph nodes.

## Discussion

In this paper, we describe, for the first time to our knowledge, the purposeful transmission of the giant Marseillevirus to a murine host. By including different routes of inoculation, our model aimed to assess the tropism, persistence and dissemination of the virus. We report a 30-day long persistence of the virus in immunocompetent rats and mice inoculated by the IP, IV and respiratory routes. The virus was able to disseminate from the peritoneum to the bloodstream as well as from the bloodstream into several deep organs. The NALT, a rodent equivalent of the human tonsils, appeared to be an important target organ after aerosol transmission, as attested by its early and lasting carriage at high viral loads as compared to other organs. The viral load, as assessed by quantification of DNA copies when possible, did not increase over the time regardless the animal model or the route of inoculation, so we cannot clearly conclude to the evidence of *in vivo* replication of the virus.

The presence of giant viruses in mammalian hosts was first suggested for mimiviruses, other giant viruses which are close relatives of marseilleviruses. Thus, Mimivirus-associated pneumonia have been described, notably in one patient from which the virus could be isolated from its broncho-alveolar fluid (Saadi et al., 2013a). Another case featuring a laboratory technician handling Mimivirus who developed unexplained pneumonia and seroconversion to Mimivirus antigens which has also been reported (Raoult et al., 2006; Saadi et al., 2013a,b). Moreover, the sero-epidemiological data show a significantly higher seroprevalence for mimivirus in pneumonia patients than in controls. Indeed, on 887 serum samples including 376 from patients with community-acquired pneumonia, and 511 from healthy control subjects, 9.66% of the first group exhibited a positive titer of antibodies to Mimivirus whereas only 2.3% of the healthy controls were positive (*p* = 0.01; La Scola et al., 2005). Moreover, Mimivirus DNA was detected by PCR in respiratory samples from a patient with hospital-acquired pneumonia (La Scola et al., 2005). However, studies using PCR assays were more difficult to conduct because of the great genetic variability of the mimiviruses genomes, a feature shared with marseilleviruses. Thus, Dare et al. screened 496 respiratory specimens from nine pneumonia patient populations for Mimivirus by qPCR, performed mainly on nasal and nasopharyngeal swabs. All the samples tested were negative (Dare et al., 2008).

The clinical data mentioned above were completed by a mouse model reproducing histologically proven pneumonia at days 3 and 7 PI in C57BL/6 and BALB/c mice respectively (Khan et al., 2007). Another giant virus, *Acanthocystis turfacea* Chlorella Virus 1, a close relative of amoeba giant viruses from the family *Phycodnaviridae*, was found in oro-pharyngeal samples from patients and was associated with a decrease in cognitive functioning (Yolken et al., 2014). A mouse model showed that digestive inoculation of the virus induced, modifications in the brain of the expression of genes involved in cognitive functions. These authors supposed that the virus was responsible for cognitive impairment, although such a hypothesis would need further investigation (Yolken et al., 2014).

In the present work, the IP model showed an early transient blood dissemination of the virus both in mice and rats, and its persistence in the spleen for at least 2 weeks. The IV model also showed that after a transient passage in the bloodstream, viable Marseillevirus was detected as much as 3 weeks later in the spleen. In the aerosolized model, the virus was detected at a higher frequency in NALT than in lung samples, especially at later time points. Interestingly, the DNA viral load at day 30 PI was 4.7 log units of viral copies per million of murine cells, in other words, not that different to the load just after aerosolization (5.9 log units of viral copies per million of murine cells). Conversely, in the lungs, the viral load regularly decreased until it was undetectable at day 30 PI. Although our results do not show a viral replication, the long persistence into the NALT of aerosol inoculated mice is congruent with the human case of Marseillevirus persistence in pharyngeal samples (Aherfi et al., 2016b).

The use of two techniques (amoebal co-culture and PCR) for detecting the virus, complemented with antibody detection assays under strict control conditions and predefined strict criteria for the PCR and serology interpretations strengthens our results. In addition, double blind reading of immunofluorescence assays was performed. This could have led to the under diagnosis of positive serological responses after aerosolization. Concerning the antibody response after parenteral inoculations, a strong concordance was obtained.

The absence of any pathological findings in the organs, including the lungs, could be due to an absence of a detectable host cellular immune response or to the invasiveness of the pathogen. However, it is not known whether immune-suppressed animals or repetitive contact may have induced some of these cases.

The presence of marseilleviruses in humans has previously been reported from different cases, including blood from healthy donors, one case of adenitis, one case of Hodgkin's lymphoma and, as a chronic carriage, in a patient with neurological symptoms (Popgeorgiev et al., 2013a,c; Aherfi et al., 2016a,b). Our results in mice and rats reinforce the hypothesis of chronic carriage. Considering the low number of proportion of positive clinical samples, either for mimiviruses or for marseilleviruses, we can hypothesize that the techniques used to detect giant viruses lack of sensitivity. There are undeniably, a lot of technique improvements that remain to do, both on culture isolation and PCR techniques for detecting giant viruses in clinical samples. Thus, the low number of viral particles combined with the lack of sensitivity of the techniques used may lead to a low number of positive samples in the samples collected. It is noteworthy that it has not been established to date that marseilleviruses can replicate in mammals, or cause a disease. However, giant viruses of amoebae, which are very distant from other viruses both by their phenotypic and genotypic features, might act on mammal cells by a different mechanism than replication. Thus, the big size of giant viruses may probably enable their ingestion by phagocytic cells, without the intervention of a specific cell receptor (Ghigo et al., 2008).

The absence of pathological findings in the tested organs points toward the healthy carriage of the virus by the host. However, further investigations should be performed to assess whether recurrent contact with Marseillevirus or if an inoculation in immunocompromised mice may favor a pathologic outcome. Although the virus was not detected in the lymph nodes in our work, it was found to be viable for as long as 2–3 weeks in the spleen after IP and IV inoculation, respectively.

Given the high prevalence of marseilleviruses in the environment, and the possibility of a long term carriage, further investigations are needed on the mechanisms used by these viruses to escape rapid destruction by immune system. It would be interesting to try culturing Marseillevirus on different professional phagocytic cells, as was performed for Mimivirus, to assess if at least some of them are permissive. To date, only amoebas are known to be a host for Marseillevirus that allow a complete lytic replication cycle. However, if humans are possible carriers of Marseillevirus, they might serve as vectors for their dissemination in the environment. In summary, this experimental model is a first step toward the assessment of Marseillevirus infection in a mammalian host. Its long persistence, especially in the NALT, merits further study to assess the possibility of a longer viral persistence and reinforces the pertinence of systematic Marseillevirus detection in subjects presenting with unexplained upper airway/pharyngeal or adenitis clinical pictures.

## Author contributions

DR, FB, and SA designed the project. CN, FB, and SA implemented the animal experiments. HL analyzed the tissue sections for anatomo-pathology. MB set the protocol of PC, LB, and SA performed the amoebal co-cultures. PJ and SA performed the PC experiments. PC, BS, and DR supervised the project. FB and SA wrote the manuscript.

### Conflict of interest statement

The authors declare that the research was conducted in the absence of any commercial or financial relationships that could be construed as a potential conflict of interest.
