# Supplementary material for: Experimental Inoculation in Rats and Mice by the Giant Marseillevirus Leads to Long-Term Detection of Virus
Source: Front Microbiol. 2018 Mar 21;9:463. doi: 10.3389/fmicb.2018.00463 (PMC5871663; doi:10.3389/fmicb.2018.00463)
Supplement: Supplementary file 1 [file Table1.DOCX]

| **IP route in mice** | | | | | | | |
| --- | --- | --- | --- | --- | --- | --- | --- |
|  |  | **PCR** | | | **Coculture** | | |
| **Mice** | **Day post inoculation** | **Spleen** | **Blood** | **Liver** | **Spleen** | **Blood** | **Liver** |
| 1 | 0 | 0 | 0 | ND | 1 | 1 | ND |
| 2 | 0 | 1 | 1 | ND | 1 | 0 | ND |
| 3 | 0 | 1 | 1 | ND | 1 | 1 | ND |
| 4 | 0 | 1 | 0 | ND | 1 | 1 | ND |
| 5 | 1 | 1 | 1 | ND | 1 | 1 | ND |
| 6 | 1 | 0 | 0 | ND | 1 | 0 | ND |
| 7 | 1 | 1 | 1 | ND | 1 | 0 | ND |
| 8 | 1 | 0 | 0 | ND | 1 | 0 | ND |
| 9 | 2 | 0 | 0 | ND | 1 | 0 | ND |
| 10 | 2 | 1 | 1 | ND | 1 | 0 | ND |
| 11 | 2 | 0 | 0 | ND | 0 | 0 | ND |
| 12 | 7 | 0 | 0 | ND | 0 | 0 | ND |
| 13 | 7 | 0 | 0 | ND | 1 | 0 | ND |
| 14 | 7 | 1 | 0 | ND | 1 | 0 | ND |
| 15 | 21 | NI | NI | ND | 0 | 0 | ND |

Suppl file 1 Summary of results from qPCR and coculture of blood and organ samples from mice inoculated with Marseillevirus by IP route; 1=Positive; 0= Negative; ND=Not Done
