# Supplementary material for: Experimental Inoculation in Rats and Mice by the Giant Marseillevirus Leads to Long-Term Detection of Virus
Source: Front Microbiol. 2018 Mar 21;9:463. doi: 10.3389/fmicb.2018.00463 (PMC5871663; doi:10.3389/fmicb.2018.00463)
Supplement: Supplementary file 2 [file Table2.DOCX]

|  |  | **PCR** | | | **Coculture** | | | **Day 0** | | **Day 1** | |
| --- | --- | --- | --- | --- | --- | --- | --- | --- | --- | --- | --- |
| **Rats** | **Day post inoculation** | **Spleen** | **Blood** | **Liver** | **Spleen** | **Blood** | **Liver** | **PCR Blood** | **CC blood** | **PCR Blood** | **CC blood** |
| 1 | 1 | 1 | 0 | 1 | 1 | 1 | 1 | 1 | 1 | 0 | 1 |
| 2 | 1 | 1 | 1 | 1 | 1 | 0 | 1 | 1 | 0 | 1 | 0 |
| 3 | 3 | 0 | ND | 0 | 0 | ND | 0 | ND | ND | ND | ND |
| 4 | 3 | 1 | ND | 1 | 1 | ND | 0 | ND | ND | ND | ND |
| 5 | 7 | 1 | 0 | 0 | 1 | 0 | 0 | 0 | 0 | 0 | 0 |
| 6 | 7 | 1 | 0 | 0 | 1 | 0 | 1 | ND | ND | 0 | 0 |
| 7 | 7 | 1 | 0 | ND | 0 | 0 | 0 | ND | ND | ND | ND |
| 8 | 7 | ND | 0 | 0 | 1 | 0 | 0 | ND | ND | ND | ND |
| 9 | 14 | 1 | 0 | 1 | 1 | 0 | 0 | ND | ND | ND | ND |
| 10 | 14 | 1 | 0 | 1 | 1 | 0 | 0 | ND | ND | ND | ND |
| 11 | 14 | 1 | 0 | 0 | 1 | 0 | 0 | ND | ND | ND | ND |
| 12 | 43 | 0 | NI | 0 | 0 | 0 | 0 | ND | ND | ND | ND |

Suppl file 2 Summary of results from qPCR and coculture of blood and organ samples from rats inoculated with Marseillevirus by IP route1=Positive; 0= Negative; ND=Not Done; CC=Coculture. NI= Not interpretable
