# Supplementary material for: Experimental Inoculation in Rats and Mice by the Giant Marseillevirus Leads to Long-Term Detection of Virus
Source: Front Microbiol. 2018 Mar 21;9:463. doi: 10.3389/fmicb.2018.00463 (PMC5871663; doi:10.3389/fmicb.2018.00463)
Supplement: Supplementary file 3 [file Table3.DOCX]

|  |  | PCR | | | | CC | | | |
| --- | --- | --- | --- | --- | --- | --- | --- | --- | --- |
|  |  | Spleen | Liver | Blood | Lung | Spleen | Liver | Blood | Lung |
| Rats |  | rate | Foie | blood | lung | rate | Foie | blood | lung |
| 1 | Day 1 | 1 | 1 | 1 | 1 | 1 | 1 | 1 | 1 |
| 2 | Day 1 | 1 | 1 | 1 | 1 | 1 | 1 | 1 | 1 |
| 3 | Day 1 | 1 | 1 | 1 | 0 | 1 | 1 | 1 | 1 |
| 4 | Day 1 | 1 | 1 | 1 | 1 | 1 | 1 | 1 | 1 |
| 5 | Day 3 | 1 | 1 | 0 | 1 | 1 | 1 | 0 | 1 |
| 6 | Day 3 | 1 | 1 | 0 | 0 | 1 | 1 | 0 | 0 |
| 7 | Day 3 | 1 | 1 | ND | 0 | 0 | 1 | ND | 0 |
| 8 | Day 7 | 1 | 1 | 0 | 0 | 1 | 1 | 0 | 0 |
| 9 | Day 7 | 0 | 0 | 0 | 0 | 0 | 0 | 0 | ND |
| 10 | Day 7 | 1 | 0 | 0 | 1 | 1 | 1 | 0 | 1 |
| 11 | Day 14 | 1 | 1 | 0 | 0 | 1 | 0 | 0 | 0 |
| 12 | Day 14 | 1 | 1 | 0 | 0 | 1 | 1 | 0 | 0 |
| 13 | Day 14 | 1 | 1 | 0 | 0 | 1 | 1 | 0 | 0 |
| 14 | Day 21 | 1 | 1 | 0 | 0 | 1 | 0 | 0 | 0 |
| 15 | Day 21 | 1 | ND | 0 | 0 | 1 | ND | 0 | 0 |
| 16 | Day 21 | ND | 1 | 0 | 0 | ND | 0 | 0 | 0 |
| 17 | Day 28 | 1 | 1 | 0 | 0 | 0 | 0 | 0 | 0 |
| 18 | Day 28 | 1 | 1 | 0 | 0 | 0 | 0 | 0 | 0 |
| 19 | Day 28 | 0 | 0 | 0 | 0 | 0 | 0 | 0 | 0 |
| 20 | Day 43 | 0 | 0 | 0 | 0 | 0 | 0 | 0 | 0 |
| 21 | Day 43 | 0 | 0 | 0 | 0 | 0 | 0 | 0 | 0 |

Suppl file 3 Summary of results from qPCR and coculture of blood and organ samples from rats inoculated with Marseillevirus by IV route; 1=Positive; 0= Negative; ND=Not Done
