# Supplementary material for: Experimental Inoculation in Rats and Mice by the Giant Marseillevirus Leads to Long-Term Detection of Virus
Source: Front Microbiol. 2018 Mar 21;9:463. doi: 10.3389/fmicb.2018.00463 (PMC5871663; doi:10.3389/fmicb.2018.00463)
Supplement: Supplementary file 4 [file Table4.docx]

| **Mice** | **Day** | **Blood**  **PCR** | **Blood**  **CC** | **Foie**  **PCR** | **Foie**  **CC** | **Rate**  **PCR** | **Rate**  **CC** | **NALT**  **PCR** | **NALT**  **CC** | **Lungs**  **PCR** | **Lungs**  **CC** |
| --- | --- | --- | --- | --- | --- | --- | --- | --- | --- | --- | --- |
| **1** | 0 | 0 | 0 | **0** | **0** | 0 | 0 | ND | ND | 1 | 1 |
| **2** | 0 | 0 | 0 | **0** | **0** | 0 | 0 | ND | ND | 1 | 1 |
| **3** | 0 | **0** | **0** | 0 | 0 | 0 | 0 | 1 | 1 | 1 | 1 |
| **4** | 0 | **0** | **0** | 0 | 0 | 0 | 0 | 1 | 1 | 1 | 1 |
|  |  |  |  |  |  |  |  |  |  |  |  |
| **5** | **1** | **0** | 0 | **0** | **0** | **0** | 0 | ND | ND | **1** | **1** |
| **6** | **1** | **0** | 0 | **0** | **0** | **0** | 0 | ND | ND | **1** | **1** |
| **7** | **1** | **0** | 0 | **0** | **0** | **0** | 0 | ND | ND | **1** | **1** |
| **8** | **1** | **0** | 0 | **0** | **0** | **0** | 0 | ND | ND | **1** | **1** |
| **9** | **1** | **0** | 0 | **0** | **0** | **0** | 0 | 1 | **1** | **1** | **1** |
| **10** | **1** | **0** | **0** | **0** | **0** | **0** | **0** | **1** | **1** | **1** | **1** |
| **11** | **1** | **0** | **0** | **0** | **0** | **0** | **0** | **1** | **1** | **1** | **1** |
| **12** | **1** | **0** | **0** | **0** | **0** | **0** | **0** | **1** | **1** | **1** | **1** |
| **13** | **1** | **0** | **0** | **0** | **0** | **0** | **0** | **1** | **1** | **1** | **1** |
| **14** | **1** | **0** | **0** | **0** | **0** | **0** | **0** | **1** | **1** | **1** | **1** |
| **15** | **1** | **0** | **0** | **0** | **0** | **0** | **0** | **1** | **1** | **1** | **1** |
| **16** | **1** | **0** | **0** | **0** | **0** | **0** | **0** | **1** | **1** | **1** | **1** |
| **17** | **1** | **0** | **0** | **0** | **0** | **0** | **0** | **1** | **1** | **1** | **1** |
| **18** | **1** | **0** | **0** | **0** | **0** | **0** | **0** | **1** | **1** | **1** | **1** |
| **19** | **1** | **0** | **0** | **0** | **0** | **0** | **0** | **1** | **1** | **1** | **1** |
|  |  |  |  |  |  |  |  |  |  |  |  |
| **20** | 7 | 0 | 0 | **0** | **0** | 0 | 0 | 1 | 1 | 1 | 1 |
| **21** | 7 | 0 | 0 | **0** | **0** | 0 | 0 | 1 | 1 | 1 | 1 |
| **22** | 7 | 0 | 0 | **0** | **0** | 0 | 0 | 1 | 1 | 1 | 1 |
| **23** | 7 | 0 | 0 | **0** | **0** | 0 | 0 | 1 | 1 | 1 | 1 |
| **24** | 7 | 0 | 0 | **0** | **0** | 0 | 0 | 1 | 1 | 1 | 1 |
| **25** | 7 | 0 | 0 | **0** | **0** | 0 | 0 | 1 | 1 | 1 | 1 |
| **26** | 7 | **0** | **0** | 0 | 0 | 0 | 0 | 1 | 1 | 1 | 1 |
| **27** | 7 | **0** | **0** | 0 | 0 | 0 | 0 | 1 | 1 | 0 | 1 |
| **28** | 7 | **0** | **0** | 0 | 0 | 0 | 0 | 1 | 1 | 1 | 1 |
| **29** | 7 | **0** | **0** | 0 | 0 | 0 | 0 | 1 | 1 | 1 | 1 |
| **30** | 7 | **0** | **0** | 0 | 0 | 0 | 0 | 1 | 1 | 1 | 1 |
| **31** | 7 | **0** | **0** | 0 | 0 | 0 | 0 | 1 | 1 | 1 | 1 |
| **32** | 7 | **0** | **0** | 0 | 0 | 0 | 0 | 1 | 1 | 1 | 1 |
| **33** | 7 | **0** | **0** | 0 | 0 | 0 | 0 | 1 | 1 | 1 | 1 |
| **34** | 7 | **0** | **0** | 0 | 0 | 0 | 0 | 1 | 1 | 0 | 1 |
| **35** | 7 | **ND** | **0** | 0 | 0 | 0 | 0 | 1 | 1 | 1 | 1 |
| **36** | 7 | **0** | **0** | 0 | 0 | 0 | 0 | 1 | 1 | 1 | 1 |
| **37** | 7 | **0** | **0** | 0 | 0 | 0 | 0 | 1 | 1 | 1 | 1 |
|  |  |  |  |  |  |  |  |  |  |  |  |
|  |  |  |  |  |  |  |  |  |  |  |  |
| **38** | **14** | **0** | 0 | **0** | **0** | **0** | 0 | **0** | **1** | **1** | **1** |
| **39** | **14** | **0** | 0 | **0** | **0** | **0** | 0 | **1** | **0** | **1** | **1** |
| **40** | **14** | **0** | 0 | **0** | **0** | **0** | 0 | **1** | **1** | **1** | **0** |
| **41** | **14** | **0** | 0 | **0** | **0** | **0** | 0 | **1** | **1** | **1** | **0** |
| **42** | **14** | **0** | 0 | **0** | **0** | **0** | 0 | **0** | **1** | **1** | **1** |
| **43** | **14** | **0** | **0** | 0 | 0 | 0 | 0 | 1 | 1 | 1 | 1 |
| **44** | **14** | **0** | **0** | 0 | 0 | 0 | 0 | 1 | 1 | 0 | 1 |
| **45** | **14** | **0** | **0** | 0 | 0 | 0 | 0 | 1 | 1 | 1 | 1 |
| **46** | **14** | **0** | **0** | 0 | 0 | 0 | 0 | 1 | 1 | 0 | 0 |
| **47** | **14** | **0** | **0** | 0 | 0 | 0 | 0 | 1 | 1 | 1 | 0 |
| **48** | **14** | **0** | **0** | 0 | 0 | 0 | 0 | 1 | 1 | 0 | 1 |
| **49** | **14** | **0** | **0** | 0 | 0 | 0 | 0 | 1 | 1 | 0 | 0 |
| **50** | **14** | **ND** | **0** | 0 | 0 | 0 | 0 | 1 | 1 | 0 | 1 |
| **51** | **14** | **0** | **0** | 0 | 0 | 0 | 0 | 1 | 1 | 0 | 0 |
| **52** | **14** | **ND** | **0** | 0 | 0 | 0 | 0 | 1 | 1 | 0 | 0 |
|  |  |  |  |  |  |  |  |  |  |  |  |
| **53** | 21 | **0** | 0 | **0** | **0** | **0** | 0 | 0 | 1 | 0 | 0 |
| **54** | 21 | **0** | 0 | **0** | **0** | **0** | 0 | 0 | 1 | 0 | 0 |
| **55** | 21 | **0** | 0 | **0** | **0** | **0** | 0 | 0 | 0 | 0 | 0 |
| **56** | 21 | **0** | 0 | **0** | **0** | **0** | 0 | 1 | 1 | 0 | 0 |
| **57** | 21 | **0** | 0 | **0** | **0** | **0** | 0 | 1 | 0 | 0 | 0 |
| **58** | 21 | **0** | 0 | **0** | **0** | **0** | 0 | 0 | 1 | 0 | 0 |
| **59** | 21 | **0** | **0** | 0 | 0 | 0 | 0 | 0 | 0 | 0 | 0 |
| **60** | 21 | **0** | **0** | 0 | 0 | 0 | 0 | 1 | 1 | 0 | 0 |
| **61** | 21 | **ND** | **0** | 0 | 0 | 0 | 0 | 0 | 1 | 0 | 1 |
| **62** | 21 | **ND** | **0** | 0 | 0 | 0 | 0 | 1 | 1 | 0 | 0 |
| **63** | 21 | **0** | **0** | 0 | 0 | 0 | 0 | 1 | 1 | 0 | 0 |
| **64** | 21 | **0** | **0** | 0 | 0 | 0 | 0 | 1 | 1 | 0 | 0 |
| **65** | 21 | **0** | **0** | 0 | 0 | 0 | 0 | 0 | 1 | 0 | 0 |
| **66** | 21 | **0** | **0** | 0 | 0 | 0 | 0 | 1 | 1 | 0 | 0 |
|  |  |  |  |  |  |  |  |  |  |  |  |
| **67** | 30 | **0** | **0** | 0 | 0 | 0 | 0 | ND | ND | 0 | 0 |
| **68** | 30 | **0** | **0** | 0 | 0 | 0 | 0 | ND | ND | 0 | 0 |
| **69** | 30 | **0** | **0** | 0 | 0 | 0 | 0 | 1 | 1 | ND | ND |
| **70** | 30 | **0** | **0** | 0 | 0 | 0 | 0 | 1 | 1 | 0 | 1 |
| **71** | 30 | **0** | **0** | 0 | 0 | 0 | 0 | 1 | 1 | 0 | 0 |

Suppl file 4 Summary of results from qPCR and coculture of blood and organ samples from mice inoculated with Marseillevirus by aerosolized route; 1=Positive; 0= Negative; ND=Not Done; CC= result from coculture
