# Supplementary figures and images for: Experimental Inoculation in Rats and Mice by the Giant Marseillevirus Leads to Long-Term Detection of Virus
Source: Front Microbiol. 2018 Mar 21;9:463. doi: 10.3389/fmicb.2018.00463 (PMC5871663; doi:10.3389/fmicb.2018.00463)

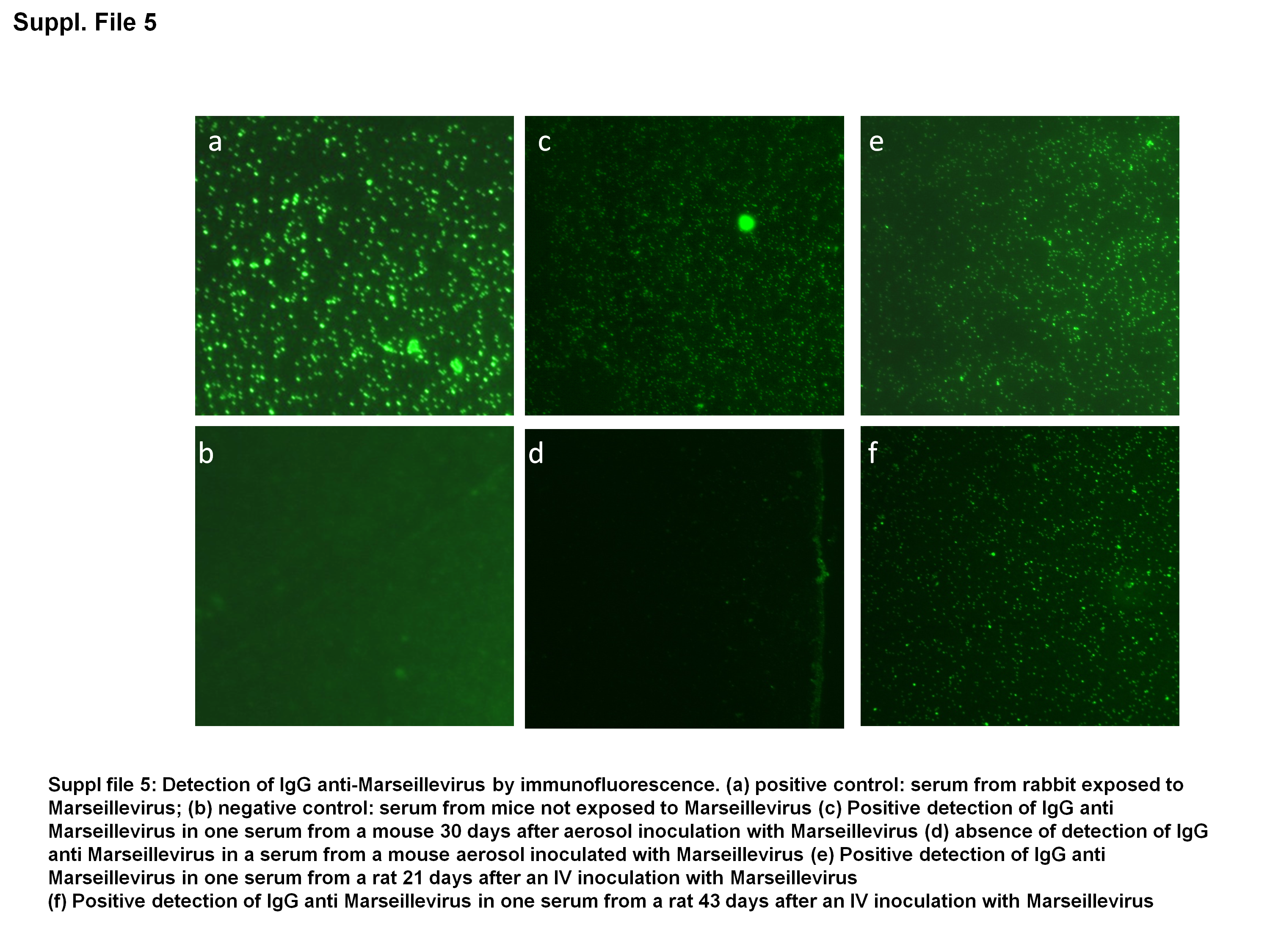

Supplement: Supplementary file 5 [file Image1.TIF]
